# Is the current UK undergraduate system providing junior doctors knowledge and confidence to manage burns? A questionnaire-based cohort study

**DOI:** 10.1186/s41038-015-0005-9

**Published:** 2015-05-28

**Authors:** Thomas I Lemon, Simon Stapley, Andrea Idisis, Ben Green

**Affiliations:** 1 North Lincolnshire and Goole NHS Trust, North Lincolnshire, UK; 2 School of Medicine, Leeds University, Leeds, UK; 3 Leeds Teaching Hospitals, Leeds, UK; 4 Postgraduate Medical Education Centre, Diana Princess of Wales Hospital, Scartho, Lincolnshire, DN332BA UK

**Keywords:** Burns, Undergraduate, Medical education

## Abstract

**Background:**

Burns are common in the UK and many of the 30,000 newly qualified doctors there will be faced with managing them in their first few years of practice. We are concerned that doctors are leaving medical school without adequate teaching on burns and therefore not prepared to manage burns competently. The aim of this study was to assess the graduating doctors self-declared knowledge of basic burns pathology as well as their knowledge and confidence in treating burns. We also wanted to assess whether students felt that their undergraduate course offered burns teaching, either formally or informally.

**Methods:**

We designed a structured questionnaire with input from two experienced final year medical students, two experienced clinicians and two sociologists. Questions were designed to be open-ended in order to facilitate varied and circumstantiated responses. Final year medical students, due to graduate in June 2014, were invited to take part in a survey with questions on burns management, first aid, pathology, and confidence. These results were then analyzed statistically.

**Results:**

Of the 300 students invited to join the survey, 244 fully completed the process, representing an 81.3% response rate. Of the respondents over one-third (35%) said they had not received any teaching on burns. And less than half (45%) said they had received formal teaching. Eighty-eight percent of students identified a burn can be caused by a dry heat source; however, 17% of students failed to acknowledge that chemicals are a recognized cause of burns. Only 32% of respondents were confident with management of a burn.

**Conclusions:**

These results suggest that there is a lack in understanding of burns management, as well as a lack of confidence in treating burns among graduating doctors. There was also a self-identified lack of teaching at an undergraduate level. These concerning results could be improved by the integration of burns into the core medical curriculum.

## Background

Burns in the UK are common with the majority of burns happening in young children and the working-age population [1]. The extent of this preventable problem is highlighted in the National Burn Care Review of 2001 that reported over 250,000 burns a year, 175,000 of which will attend emergency departments [2].

In the UK, plastic surgery is the speciality primarily responsible for the management of burns. Despite the extent of the problem, plastic surgery, and therefore burns management, is not routinely taught in the UK core medical undergraduate curriculum [3]. The opportunity for graduating doctors to learn about the assessment and management of burns is therefore woefully limited.

This is further evidenced by studies, which have suggested that there is insufficient teaching on burns management at UK medical schools. The UK is responsible for over 30,000 graduating doctors each year from its 33 medical schools [4]. Previous studies suggest that only 13% of medical schools educate their students on burns management in a structured way, and of the 29 medical schools surveyed, the maximum time allowance for burns teaching was found to be limited to a total of just 4 h [5].

The absence of burns teaching in UK medical schools may be in part related to the disparities in current evidence and guidelines regarding the most appropriate initial management. This is further compounded by a wide variation in the pre-hospital management of burns and the absence of national pre-hospital guidance on initial burns management [5,6].

The aim of this study was to assess UK-based graduating medical students’ self-declared knowledge of basic burn pathology as well as appropriate burns management. We also intend to discover whether students feel that they are receiving teaching regarding burns and their management. We suspect that a lack of undergraduate education to UK medical students is resulting in the graduating doctors beginning work lacking the basic understanding of burns and burns management and therefore are not confident to look after burns patients. We also suspect that the current cohort of graduating doctors is likely to agree with the literature regarding insufficient burns education.

## Materials and methods

### Participants

The questionnaire was delivered either electronically or via postal service to 300 randomly selected individuals subscribing to the United Kingdom Medical Students’ Association (UKMSA) and Meducation. Individuals were identified using Research Randomizer, a program that utilizes a JavaScript random number generator to produce customized sets of random numbers. This was chosen as the UKMSA database holding information on subscribers subject to constant change as new members join and therefore reflects a current cohort of UK medical students. The selected cohort of respondents contained final-year medical students from 23 of the UK’s 33 medical schools.

### Questionnaire

We designed a structured questionnaire (available on request) with input from two experienced final-year medical students, two experienced clinicians, and two sociologists. Questions were designed to be open-ended in order to facilitate varied and circumstantiated responses. This broad approach was intended to capture subject-rich information regarding the respondents’ knowledge and confidence levels relating to burns management without leading respondents’ answers. A limited number of questions were closed with designated response categories in order to facilitate the collation of anonymous respondent demographic information.

The questionnaire contained eight questions pertaining to both the pathology process and clinical management of burns. We addressed the following items: whether teaching had been received during the 5-year undergraduate programme, knowledge of the burns first-aid, knowledge of basic burn pathology, and whether or not, at the point of graduation, the respondent felt confident in managing an acute burn. Because the majority of questions were in an open-ended format, thereby allowing multiple responses from each participant, we grouped responses according to consensus into broader, more inclusive categories for discussion purposes. Responses were analysed and sorted into largely positive or negative categories, forming the basis of numerical analysis. These were set by a pre-determined threshold as agreed by our clinician advisors, as acceptable and appropriate management, or inappropriate management. Ethnographic methods of conversation analysis as applied to literature were also used by the sociologists to the style of response students gave to enhance the analysis of this process.

Our inclusion criteria and guidelines for the aspects studied were as follows:

Teaching—Formal teaching was described as a lecture or seminar given as part of a formal teaching or curriculum programme. Informal teaching was said to cover bedside teaching encounters at the moment of patient contact (we agreed that formal bedside teaching encounters could be classed as part of a structured curriculum) or brief ward-based discussions.

Pathology—The basic pathology knowledge was simply split into “cause” and “effect.” Respondents were asked to identify causative agents in the matter of burns. Regarding effect, respondents were asked about severity of burns as applied to skin layers.

First aid—First-aid management was taken from the teaching of National Health Service (NHS)-led burn management study days and the advice given to health professionals at these events. There are two key points frequently mentioned at these events: the use of cool water for a certain period and the use of cling film as a suitable product in the immediate burns management.

### Statistical analysis

Respondent’s answers were grouped and are presented in number and percentage.

## Results

### Participant demographics

Of the 300 students invited to join the survey, 244 fully completed the process, representing 81.3% response rate. All 244 respondents stated that they felt burns management was an important competency in a graduating doctor. All respondents had received all their undergraduate training in the UK, and all respondents had taken the 5-year course.

### Burns teaching received

**Figure 1:**
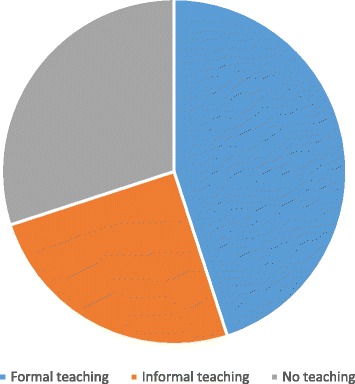
Figure demonstrating graduating doctors’ exposure to teaching on burns in the UK.

A total of 110 (45%) of the respondents had received formal teaching, a further 49 (20%) had received informal teaching, and 85 (35%) of final-year medical students in the UK stated they had received no teaching relating to burns (Figure 1).

### Core pathology knowledge of graduating UK doctors

Two hundred and fifteen (88%) of the students identified burn can be caused by a dry heat source. Forty-one (17%) students failed to acknowledge that chemicals are a recognized cause of burns.

Eighty-eight (36%) students correctly identified skin layers affected by varying severity of burns. Further to this, a total of 20 (8%) students incorrectly believed that blisters occur after the scar process had been completed.

### Burns first-aid knowledge of graduating UK doctors

**Figure 2:**
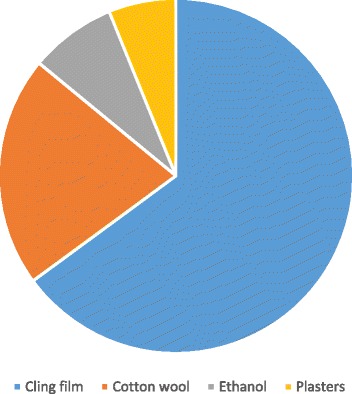
Figure demonstrating graduating doctors’ knowledge of burns first-aid.

Regarding first aid, 181 (74%) identified cling film as being the most important tool out of the items listed; however, 24 (10%) stated that cotton wool was important, 22 (9%) advised the use of ethanol, and 17 (7%) recommended the use of plasters (Figure 2).

One hundred and sixty-three (67%) said the first action they would take in a first-aid situation would be to apply water for between 10 and 30 min. Seventeen (7%) said they would use butter.

Only 7 (3%) of all respondents correctly identified de-roofing as an important management for blisters.

### UK medical graduates’ confidence in management

When asked if they would feel confident in managing a burn patient, only 78 (32%) students said they would be confident.

## Discussion

This study importantly reflects opinions of graduating students to a peer body, rather than an employer or service moderator. Our study clearly highlights that through lack of education, there is a lack of ability and confidence from newly qualified junior doctors to manage the burned patient. To provide care to a burned patient is at the core of services a newly qualified doctor should be able to provide an employer and, more importantly, a patient.

Ergo and Estela previously stated that 13% of medical undergraduates have received structured teaching; we have found a higher rate of 45% [4]. Bedside teaching encounters are of importance in analysing components in structured teaching, and this was not analysed in this study. Furthermore, our study only included medical students due to graduate in July 2014. The result of this is more clinical contact time and hence a higher likelihood of having had contact with burns. However, anything less than 100% is less than satisfactory, and our results concur with Ergo and Estela in highlighting this concerning deficit.

The lack of teaching goes hand in hand with the lack of first-aid knowledge, the confidence in management and the lack of basic pathology knowledge. All our results point to a serious neglect in the undergraduate curriculum.

We argue that undergraduate medical courses in the UK currently lack fundamental burns teaching, meaning that many graduates are unprepared and unconfident in the management of burns. The poor results seen in our survey regarding first-aid management of burns is a reflection of this gap in education. Students are rarely given the opportunity to get involved with burns cases during clinical attachments. Integration of the clinical speciality managing burns into the undergraduate curriculum is vital in order for medical undergraduates to learn how to assess and manage burns [3,7].

Even where there is some potential for exposure to burns care and education, students felt that the clinical rotations in the speciality involved were inadequate [8,9].

There have been some proposed measures to integrate burns into the curriculum, either during a casualty rotation or even as part of psychiatry education involving the sequela of burns [3,10-12]. Although there are few examples, the University of East Anglia has demonstrated successful integration of the clinical speciality covering burns into their existing curriculum and they hope to build some specific burn focus into their final-year programme, thus demonstrating that this indeed is feasible [3,11,13]. As the use of simulation in medical education becomes increasingly prevalent, there has been some research into simulation specifically for burns education [14-16]. This offers a potential route for building confidence if real-life clinical exposure cannot be guaranteed.

Due to the open-ended nature of our survey, it is beyond the scope of our results to try and compare results from the different sub-groups. It is difficult to say from these results if any particular teaching method would yield better results, either in knowledge or confidence. There is an opportunity to further assess how teaching will affect the students’ self-identified knowledge and confidence with a more focused survey.

## Conclusions

These results are alarming. While it can be argued that due to the ever-expanding scope of medicine, we need to ensure that the undergraduate curriculum is both focused and excludes unnecessary material, the knowledge of assessment and management of burns is important and should be integrated into the undergraduate curriculum in the UK. At present, many graduates in the UK have neither the knowledge nor the confidence to manage burns.

### Clinical implication

Graduates in the UK frequently work in front-line acute medical and surgical departments. A paucity of teaching in burns management leaves a void in timely service delivery.
